# First person – Ting Deng

**DOI:** 10.1242/dmm.052551

**Published:** 2025-08-19

## Abstract

First Person is a series of interviews with the first authors of a selection of papers published in Disease Models & Mechanisms, helping researchers promote themselves alongside their papers. Ting Deng is first author on ‘ Loss of *Drosophila* ribosomal protein S6 kinase II causes mitochondrial dysfunction and cell death’, published in DMM. Ting conducted the research described in this article while a PhD student in Miguel Martins's lab at University of Cambridge, Cambridge, UK. She is now a PhD student in the lab of Heike Laman at University of Cambridge, investigating how RSK kinases regulate mitochondrial health and their role in neurodegenerative diseases.


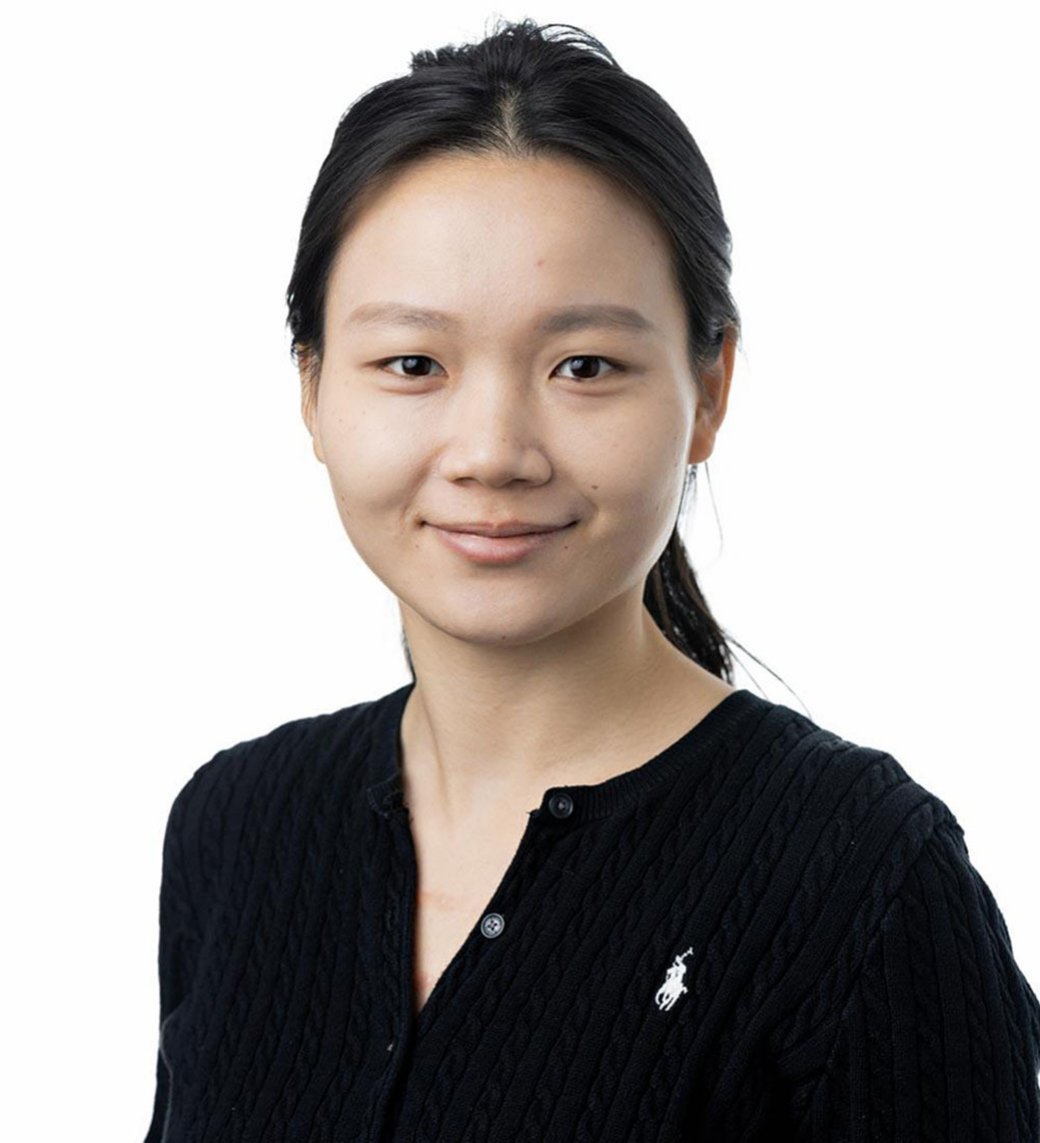


**Ting Deng**

**Who or what inspired you to become a scientist?**

I've always been curious about how the world works as I was growing up, especially the complexity of living organisms. During my high school, I became fascinated by biology and the potential to make discoveries that could improve people's lives. A key inspiration was seeing how scientific research can uncover the mechanisms behind diseases and lead to new treatments. This motivated me to pursue a career where I can contribute to advancing knowledge and ultimately help patients.

**What is the main question or challenge in disease biology you are addressing in this paper? How did you go about investigating your question or challenge?**

The central question we aimed to address is how mitochondrial integrity is regulated in energy-demanding tissues such as the brain and muscle, and whether dysregulation of this process contributes to neurodegenerative disease mechanisms. Although mitochondrial dynamics are known to be vital for cell function and survival, the upstream signalling pathways that maintain mitochondrial health under physiological and pathological conditions remain incompletely understood.

To investigate this, we focused on the role of the Ribosomal S6 kinase (RSK) family, which are downstream effectors of ERK signalling – a pathway implicated in both metabolism and neurodegeneration. Using a combination of siRNA knockdown in human cell lines and CRISPR-mediated gene knockout in *Drosophila melanogaster*, we systematically explored the impact of RSK loss on mitochondrial morphology, function and tissue viability. Through phenotypic analyses, confocal imaging and rescue experiments with human RSK4, we demonstrated a conserved role for RSKs in maintaining mitochondrial integrity. Our work bridges ERK–RSK signalling with mitochondrial dynamics and offers new insight into how its disruption may contribute to neurodegenerative disease.

…this research could contribute to strategies that protect high-energy tissues and improve patient outcomes in neurodegenerative disorders

**How would you explain the main findings of your paper to non-scientific family and friends?**

Our body needs energy to function, especially in parts like the brain and muscles. Inside our cells, tiny engines called mitochondria make this energy. We found that a group of proteins called RSKs help keep these engines working properly. When these proteins are missing, the engines break down, and the cells start to get sick and die. This can be especially harmful in tissues that need a lot of energy, like the brain. We saw this happen in both human cells and fruit flies. But when we added back one of the human RSK proteins and fruit fly S6kII, the cells and tissues got better. This means RSKs are really important for keeping our cells healthy. Our work could help us understand why mitochondria stop working in brain diseases, and how we might fix them.

**What are the potential implications of these results for disease biology and the possible impact on patients?**

Our findings suggest that RSK proteins play a crucial role in maintaining the health of mitochondria, which are essential for energy production in cells. Since mitochondrial dysfunction is linked to many neurodegenerative diseases, understanding how RSKs regulate mitochondria could reveal new targets for therapy. If we can develop treatments that boost or mimic RSK function, it may help prevent or slow down tissue damage in diseases like Parkinson's disease or Alzheimer's disease. Additionally, this work improves our understanding of how signalling pathways control cell health, offering broader insights into disease mechanisms. Ultimately, this research could contribute to strategies that protect high-energy tissues and improve patient outcomes in neurodegenerative disorders.

**Figure:**
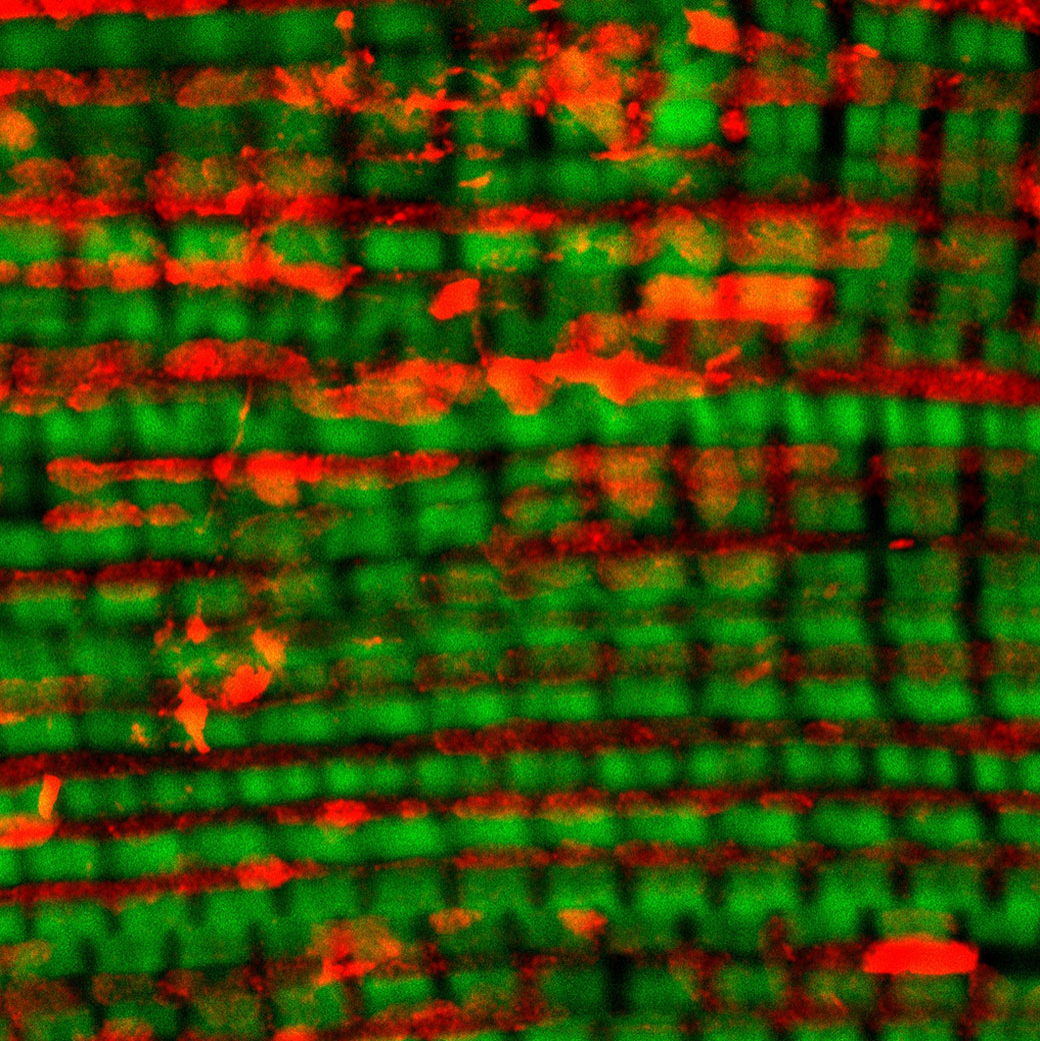
ATP5A (green) and phalloidin (red) immunostaining in the muscle of S6kII knockout *Drosophila*.

**Why did you choose DMM for your paper?**

We chose DMM because it focuses on research that connects basic biological mechanisms to human disease, which aligns perfectly with our study on mitochondrial dysfunction in neurodegeneration. DMM has a strong reputation for publishing high-quality, mechanistic studies using model organisms like *Drosophila*, which was central to our work. The journal's interdisciplinary readership ensures that our findings will reach scientists working on both fundamental biology and disease-related questions. Additionally, DMM's emphasis on translational relevance makes it an ideal platform to highlight how our discoveries could inform future therapeutic strategies.

**Given your current role, what challenges do you face and what changes could improve the professional lives of other scientists in this role?**

As a PhD student, one challenge I face is managing the balance between conducting experiments, analysing data and learning new skills, all while meeting project milestones. The pressure to produce meaningful results can sometimes feel overwhelming, especially when experiments don't go as planned. Additionally, limited access to funding and resources can restrict the scope of some research projects. To improve the professional lives of PhD students, more structured mentorship and mental health support would be helpful. Increased opportunities for skill development and clearer guidance on career paths could also make a big difference. Lastly, fostering a collaborative and supportive lab environment helps reduce stress and encourages growth.

The pressure to produce meaningful results can sometimes feel overwhelming, especially when experiments don't go as planned.

**What's next for you?**

Next, I plan to build on my current research by further investigating the molecular mechanisms through which RSK proteins regulate mitochondrial function, particularly in neuronal cells. I aim to deepen my understanding of how these pathways contribute to neurodegeneration. I also hope to collaborate with other researchers to help translate our findings into potential therapeutic strategies. In the future, I plan to continue my research on neurodegeneration as a postdoctoral fellow, preparing for a long-term career in this field.

**Tell us something interesting about yourself that wouldn't be on your CV**

Outside of the lab, I really enjoy cooking and experimenting with new recipes. I find it's a great way to relax and be creative, and it helps me manage stress and feelings of depression. Cooking also teaches me patience and attention to detail, qualities that are valuable in both life and science. Plus, sharing meals with friends and family always brings a sense of happiness and balance to my life.

## References

[DMM052551C1] Deng, T., Kalmar, L., Loh, S., Pardo, O. E. and Martins, L. M. (2025). Loss of *Drosophila* ribosomal protein S6 kinase II causes mitochondrial dysfunction and cell death. *Dis. Model. Mech*. 18, dmm052374. 10.1242/dmm.052374 40827382 PMC12403519

